# Aberrant hippocampal neurogenesis contributes to epilepsy and associated cognitive decline

**DOI:** 10.1038/ncomms7606

**Published:** 2015-03-26

**Authors:** Kyung-Ok Cho, Zane R. Lybrand, Naoki Ito, Rebecca Brulet, Farrah Tafacory, Ling Zhang, Levi Good, Kerstin Ure, Steven G. Kernie, Shari G. Birnbaum, Helen E. Scharfman, Amelia J. Eisch, Jenny Hsieh

**Affiliations:** 1 Department of Molecular Biology and Hamon Center for Regenerative Science and Medicine, UT Southwestern Medical Center, Dallas, Texas 75390, USA; 2 Department of Pharmacology, School of Medicine, The Catholic University of Korea, Seoul 137-701, South Korea; 3 Department of Psychiatry, UT Southwestern Medical Center, Dallas, Texas 75390, USA; 4 Department of Clinical Research, Oriental Medicine Research Center, Kitasato University, Tokyo 108-8641, Japan; 5 Department of Neurology & Neurotherapeutics, UT Southwestern Medical Center, Dallas, Texas 75390, USA; 6 Jan and Dan Duncan Neurological Research Institute at Texas Children’s Hospital and Baylor College of Medicine, Houston, Texas 77030, USA; 7 Department of Pediatrics, Columbia University, New York, New York 10027, USA; 8 The Nathan Kline Institute for Psychiatric Research and NYU Langone Medical Center, Orangeburg, New York 10962, USA

## Abstract

Acute seizures after a severe brain insult can often lead to epilepsy and cognitive impairment. Aberrant hippocampal neurogenesis follows the insult but the role of adult-generated neurons in the development of chronic seizures or associated cognitive deficits remains to be determined. Here we show that the ablation of adult neurogenesis before pilocarpine-induced acute seizures in mice leads to a reduction in chronic seizure frequency. We also show that ablation of neurogenesis normalizes epilepsy-associated cognitive deficits. Remarkably, the effect of ablating adult neurogenesis before acute seizures is long lasting as it suppresses chronic seizure frequency for nearly 1 year. These findings establish a key role of neurogenesis in chronic seizure development and associated memory impairment and suggest that targeting aberrant hippocampal neurogenesis may reduce recurrent seizures and restore cognitive function following a pro-epileptic brain insult.

## Abstract (web summary)

Aberrant hippocampal neurogenesis often occurs after acute seizures that produce epilepsy and cognitive impairment but the role of neurogenesis in the development of epilepsy is unclear. Here the authors suppress adult neurogenesis in mice preceding seizures and show that it reduces subsequent chronic seizure frequency and epilepsy-associated cognitive decline.

## Graphical abstract

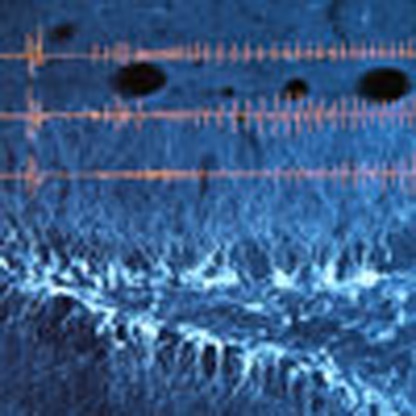

Epilepsy is a group of neurological disorders identified by unprovoked recurrent seizures^1^. Temporal lobe epilepsy (TLE) is the most common type of adult epilepsy and is characterized by seizures that initiate locally and spread throughout the entire brain^1^,^2^. It is a devastating disease, still without cure and often without effective treatment^2^. The reason why most current drug treatments do not stop the disease, but merely control convulsive seizures, may be due to a limited understanding of the basic mechanisms underlying epilepsy development (‘epileptogenesis’)^1^. Notably, acquired epilepsies due to stroke, head trauma, brain tumours, febrile seizures or status epilepticus arise after several months to years^3^. This long latency provides a possible window for therapeutic intervention. During this latent period before the onset of spontaneous recurrent seizures (SRS), a variety of cellular changes can occur in the hippocampus, that is, neuronal loss, reactive gliosis, inflammation and neurogenesis^3^. While each factor has been intensely investigated, it is not clearly known which of these alterations are critically important for development of the disease^1^,^3^.

Hippocampal neurogenesis occurs throughout life in a wide variety of mammalian species, including humans and non-human primates^4^,^5^. After most physiological and pathophysiological stimuli, adult hippocampal neurogenesis appears to be necessary and beneficial^5^,^6^,^7^,^8^,^9^, thus providing evidence for its therapeutic potential. In contrast, epileptic seizures lead to aberrant hippocampal neurogenesis, including increased proliferation of neural progenitors, production of ectopic granule cells (EGCs), mossy fibre sprouting (MFS), neuronal hypertrophy and persistence of hilar basal dendrites on adult-generated granule neurons^10^,^11^,^12^. Despite these observations, direct evidence for the role of aberrant neurogenesis in epilepsy is lacking. Past reports using nonspecific pharmacological agents suggest that inhibiting adult hippocampal neurogenesis after acute seizures leads to reduced seizures^13^,^14^, while other studies indicate that blocking adult neurogenesis via low-dose irradiation does not alter the stepwise progression of kindling^15^ or even slightly accelerated it^16^. Studies focusing on the manipulation of MFS showed that rapamycin treatment successfully inhibited MFS but produced controversial results in regards to the development of epilepsy^17^,^18^. In addition, granule neurons arising after an epileptic stimuli display variable levels of excitability^19^,^20^,^21^,^22^,^23^,^24^,^25^. Hilar EGCs receive more excitatory input and increase hippocampal excitability^20^,^21^,^22^,^23^, whereas adult-generated neurons in the granule cell layer are reported either to receive excessive excitatory input^19^,^25^ or show decreased excitability^24^. In spite of these mixed findings, a recent study showed that conditional deletion of phosphatase and tensin homologue in as little as 9% of postnatally generated granule neurons was sufficient to cause spontaneous seizures in mice^12^. However, it remains unclear whether adult-generated neurons play an essential or contributory role in the development of epilepsy. Therefore, it is necessary to define the function of adult neurogenesis in epileptogenesis to develop therapeutic interventions.

In addition to epileptogenesis, epilepsy-associated co-morbidities such as cognitive deficits often manifest in epilepsy patients^26^. Since adult hippocampal neurogenesis plays a crucial role in learning and memory^5^,^9^, seizure-induced neurogenesis may be a target to treat memory impairment in epilepsy. For instance, pharmacological modulation of seizure-induced hippocampal neurogenesis by valproic acid (VPA) or endoneuraminidase restored hippocampal-dependent memory function^27^,^28^. While these studies are consistent with the involvement of adult-generated neurons in epilepsy-associated cognitive function, these drugs may also have other cellular targets that contribute to the behavioural changes in epileptic animals. Thus, more selective manipulation of adult neurogenesis is warranted to link adult-generated neurons with epilepsy-associated cognitive function.

Here using the pilocarpine mouse model of TLE, we sought to define the role of aberrant neurogenesis in epilepsy and associated cognitive function. Using a genetic approach to inducibly suppress adult neurogenesis, we show that the ablation of neurogenesis before acute seizures reduces chronic seizure frequency, but does not completely impede epilepsy development. Even with near-complete ablation of neurogenesis pre- and post-acute seizures, there are still recurrent seizures suggesting adult neurogenesis contributes to epilepsy, but is not strictly required. Importantly, mice lacking aberrant neurogenesis show normal hippocampal-dependent novel object (NO) location recognition. Finally, the frequency of spontaneous seizures continues to be suppressed by a single ablation of neurogenesis even when evaluated at nearly 1 year after pilocarpine injection, reinforcing our hypothesis of the pro-epileptogenic role of aberrant neurogenesis and making it a promising target for permanently reducing seizures. These findings highlight the major role of adult neurogenesis in TLE and associated cognitive decline. These results also support the idea that adult neurogenesis is one of the many contributing factors to TLE.

## Results

### Genetic ablation of adult-generated granule neurons

**Figure 1:**
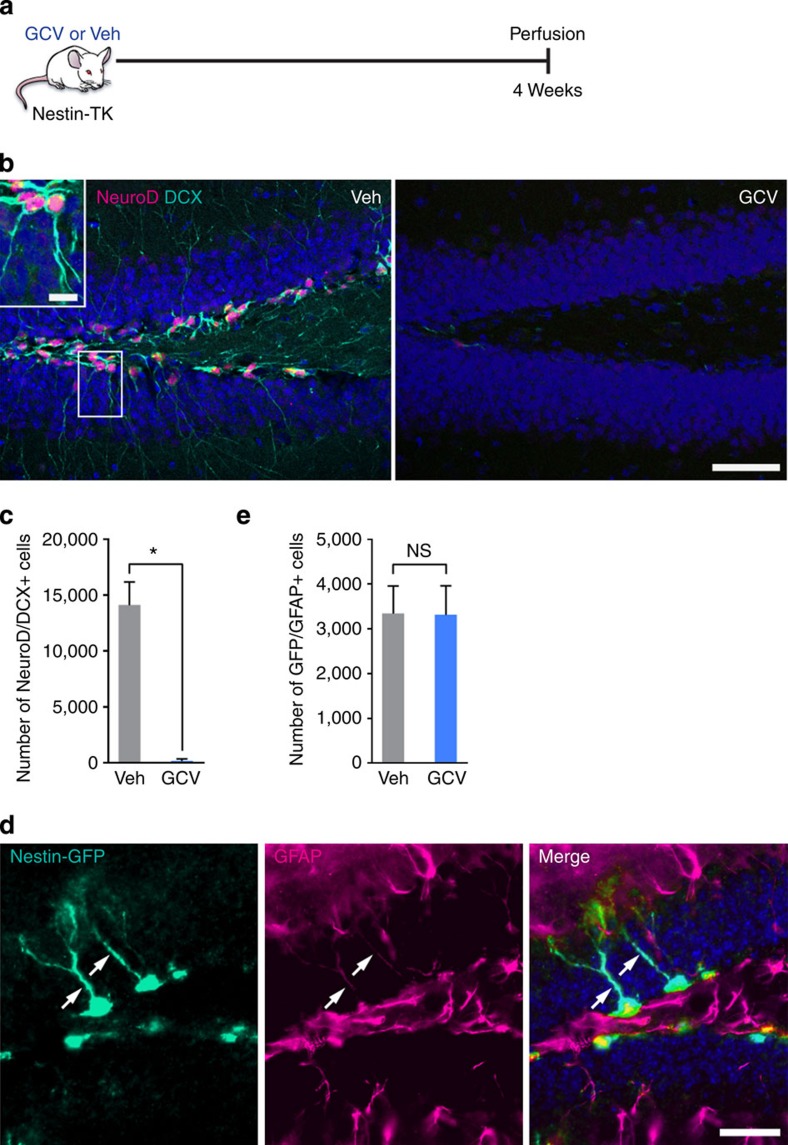
Genetic ablation of adult-born granule neurons. (**a**) Time line showing the experimental design. (**b**) Representative confocal images from three independent experiments showing dentate gyrus DCX immunostaining in mice treated with either Veh or GCV for 4 weeks. Scale bar, 50 μm. Inset shows the cells co-localized with NeuroD and DCX. Scale bar, 10 μm. (**c**) A graph showing the number of NeuroD/DCX-expressing newborn neurons in the dentate gyrus in Veh (*n*=4) and GCV group (*n*=5). Mann–Whitney *U*-test, *P*=0.016, *U*<0.001. (**d**) Representative confocal images of hippocampal neural stem cells co-expressing Nestin-GFP and GFAP out of three independent experiments. Arrows indicate representative merged cells. Scale bar, 20 μm. (**e**) A graph showing the number of GFP/GFAP-positive neural stem cells in Veh and GCV groups (*n*=6 per group). Student’s *t*-test, *P*=0.976, *t*(10)=0.031. Data presented as mean±s.e.m. *P<0.05. NS, not significant.

To investigate the role of aberrant neurogenesis in the generation of recurrent seizures, we took advantage of Nestin-δ-HSV-thymidine kinase-EGFP (Nestin-TK) transgenic mice to achieve ablation of adult hippocampal neurogenesis. This genetic model selectively ablates dividing neural stem/progenitors on ganciclovir (GCV) administration without affecting glial and endothelial cells^29^,^30^, giving us specificity superior to brain irradiation or anti-mitotic agents^13^,^15^. With 4 weeks of GCV treatment, we confirmed that NeuroD/doublecortin (DCX)-positive late-stage progenitors and neuroblasts were absent and the total number of DCX-expressing cells was decreased by more than 98% in the dentate gyrus (Fig. 1a–c, Supplementary Fig. 1). Moreover, we found no difference in the number of Nestin-TK green fluorescent protein (GFP)/glial fibrillary acidic protein (GFAP)-positive hippocampal neural stem cells between vehicle (Veh)- and GCV-treated groups, consistent with previous literature^7^,^30^ (Fig. 1d,e). Taken together, these data confirm successful ablation of adult-generated neurons within the hippocampus.

### Ablation of adult hippocampal neurogenesis reduces SRS

**Figure 2:**
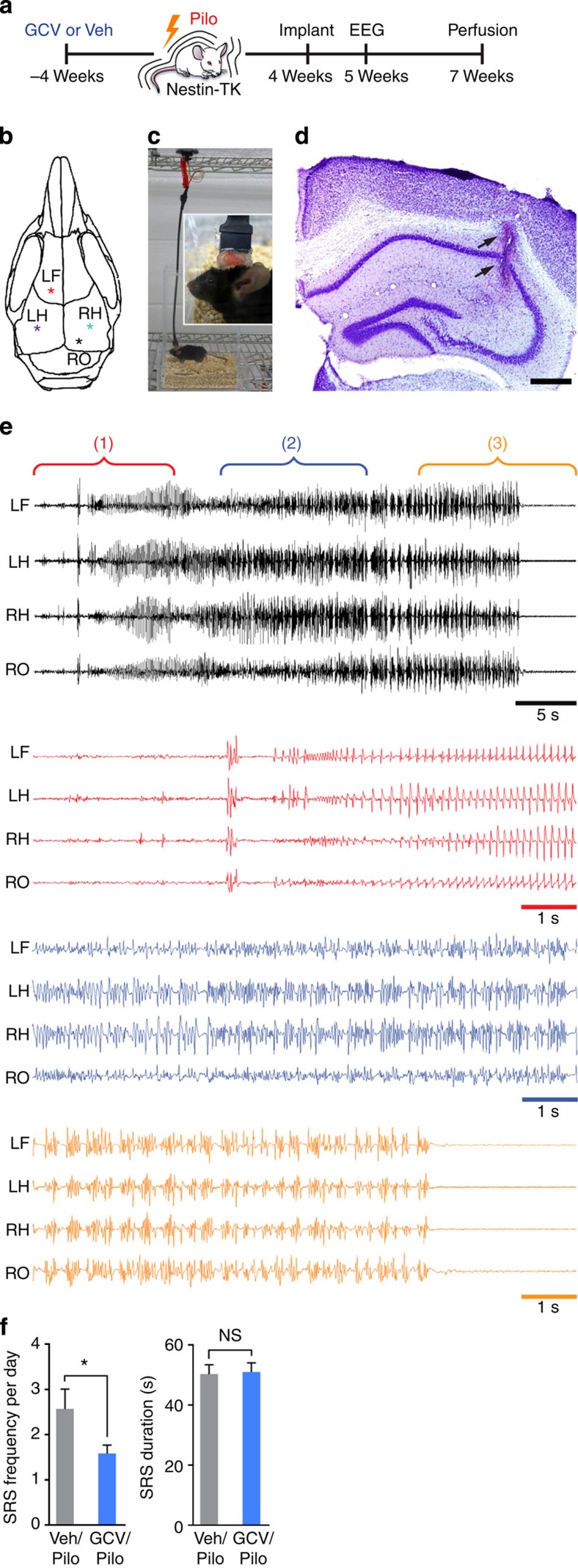
Ablation of neurogenesis reduces spontaneous seizures. (**a**) Time line showing the experimental design. (**b**) Two subdural screws and two bipolar hippocampal in-depth electrodes were implanted to record EEG. LF, left frontal screw; LH, left hippocampal depth electrode; RH, right hippocampal depth electrode; RO, right occipital screw. (**c**) Video/EEG monitoring was performed on a freely moving mouse with a tethered system. (**d**) A representative Nissl image from four independent experiments showing a hippocampal electrode track (arrows). Scale bar, 500 μm. (**e**) A representative EEG trace from eight independent experiments showing generalized seizure activity. Details are presented as initial (1), middle (2) and end sections (3). (**f**) Graphs showing the frequency and duration of SRS of GCV-treated mice (*n*=18) and Veh-treated mice (*n*=15). Student’s *t*-test, *P*=0.037, *t*(31)=2.185 for the left graph; Student’s *t*-test, *P*=0.875, *t*(31)=−0.159 for the right graph. Data presented as mean±s.e.m. *P<0.05. NS, not significant.

To examine the role of aberrant hippocampal neurogenesis in epilepsy, Nestin-TK mice were treated with Veh or GCV for 4 weeks, at which point they were injected with pilocarpine to establish chronic epilepsy (Fig. 2a). We then assessed the functional impact of ablation of neurogenesis on SRS 5 weeks after pilocarpine using continuous video/electroencephalogram (EEG) monitoring for 2 weeks. Two cortical epidural and two hippocampal depth electrodes were implanted 4 weeks after acute seizures and the number of generalized seizures, defined as simultaneous seizure activity in all four channels, was determined for 2 weeks in freely moving mice (Fig. 2b–e). Ablation of neurogenesis resulted in an ~40% reduction in SRS frequency, although SRS duration for each seizure was unaffected (Fig. 2f), suggesting that neurons born before acute seizure activity contribute to the development of recurrent seizures, but are not essential for epilepsy development.

**Figure 3:**
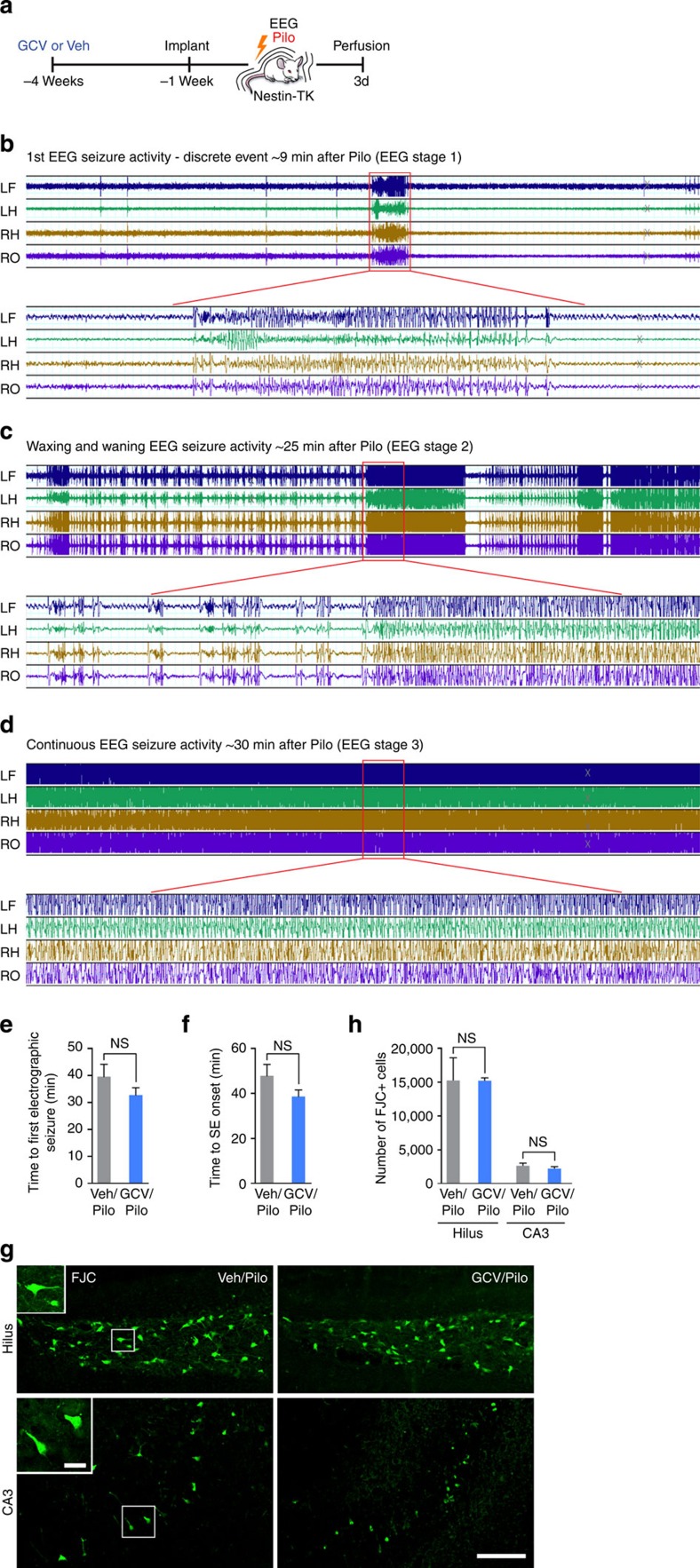
Ablating neurogenesis does not affect acute seizure severity (**a**) Experimental time line. After 4 weeks of GCV or Veh treatment, video/EEG was recorded from 1 h before pilocarpine (Pilo) injection to 3 days after acute seizures. (**b**–**d**) Representative EEG traces from two independent experiments showing EEG stage 1, stage 2 and stage 3, respectively. (**e**) A graph showing time to the first EEG seizure, which was not different between Veh/Pilo (*n*=10) and GCV/Pilo groups (*n*=13). Mann–Whitney *U*-test, *P*=0.457, *U*=53.000. (**f**) A graph showing time to status epilepticus (SE) between Veh- and GCV-treated groups (*n*=10 for Veh/Pilo, *n*=13 for GCV/Pilo). Mann–Whitney *U*-test, *P*=0.154, *U*=42.000. (**g**) Representative microscopic images from four independent experiments showing degenerating neurons labelled with FJC after acute seizures. FJC-positive cells were observed in the hilus and the CA3 subregion of the hippocampus in both Veh/Pilo and GCV/Pilo groups. Scale bar, 100 μm. Insets show typical FJC-positive cells in the hilus and the CA3 subregion. Scale bar, 20 μm. (**h**) A graph showing the number of FJC-positive cells in the hilus and the CA3 subregion of the hippocampus between Veh/Pilo (*n*=4) and GCV/Pilo (*n*=5) groups. Mann–Whitney *U*-test, *P*=1.000, *U*=10.000 for the hilar analysis; Mann–Whitney *U*-test, *P*=0.327, *U*=6.000 for the CA3 analysis. Data presented as mean±s.e.m. NS, not significant. LF, left frontal; LH, left hippocampal; RH, right hippocampal; RO, right occipital.

Next we examined the effects of ablated neurogenesis on acute seizures to exclude the possibility that a less severe period of initial seizures in the GCV/Pilo group contributed to a reduction in SRS. We recorded EEG seizure activity from 1 h before to 3 days after pilocarpine injection (Fig. 3a). Nestin-TK mice in both Veh/Pilo and GCV/Pilo groups showed a similar onset and progression of acute seizure activity from discrete events to continuous seizures, with no difference in time to the first EEG seizure or to status epilepticus (Fig. 3b–f). Consistent with no difference in acute seizure severity between the two groups, we also stained Fluoro-Jade C (FJC), a marker for degenerating neurons, for histologic confirmation of neuronal cell death after acute seizures. We found the number of FJC-positive cells in the hilus and the CA3 subregion of the hippocampus was comparable between Veh/Pilo and GCV/Pilo groups at 3 days after pilocarpine injection (Fig. 3g,h).

**Figure 4:**
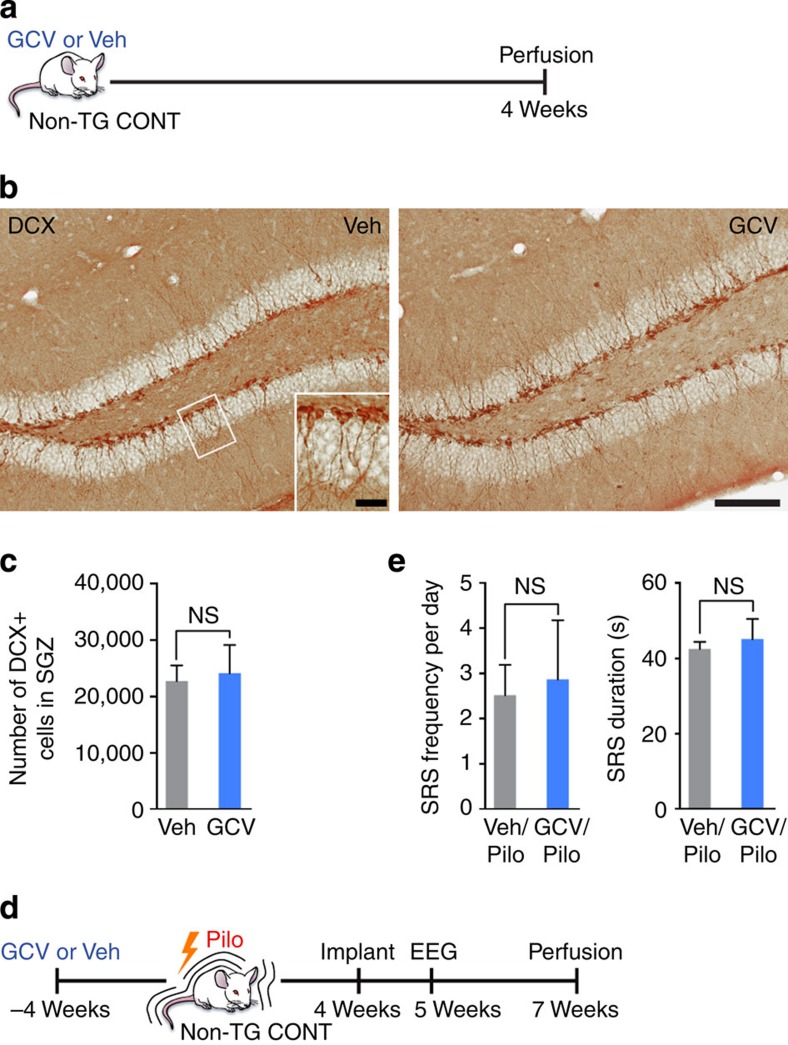
No off-target effects of GCV administration. (**a**) Experimental time line. Non-transgenic control mice without thymidine kinase (Non-TG CONT) were administered GCV or Veh for 4 weeks. **(b**) Representative microscopic images from four independent experiments showing DCX immunoreactivity between Veh and GCV groups. Scale bar, 100 μm. Inset shows typical DCX-positive cells. Scale bar, 20 μm. (**c**) A graph showing the number of DCX-expressing cells in the subgranular zone (SGZ) between Veh and GCV groups (*n*=3 per group). Student’s *t*-test, *P*=0.818, *t*(4)=−0.246. (**d**) Time line to show experimental design. (**e**) Graphs showing the frequency and duration of SRS between Veh/Pilo (*n*=8) and GCV/Pilo groups (*n*=6). Student’s *t*-test, *P*=0.803, *t*(12)=−0.255 for the left graph; Student’s *t*-test, *P*=0.664, *t*(6.243)=−0.456 for the right graph. Data presented as mean±s.e.m. NS, not significant.

As an additional control experiment, we examined the potential side effects of GCV by counting DCX-expressing cells in non-transgenic control mice without thymidine kinase (Fig. 4a–c). GCV administration had no effects on DCX-expressing cell number in the dentate gyrus, SRS frequency or SRS duration in non-transgenic control mice (Fig. 4b–e). Together these results demonstrate that adult-generated neurons promote the development of epilepsy.

### Ablating adult neurogenesis alleviates aberrant neurogenesis

**Figure 5:**
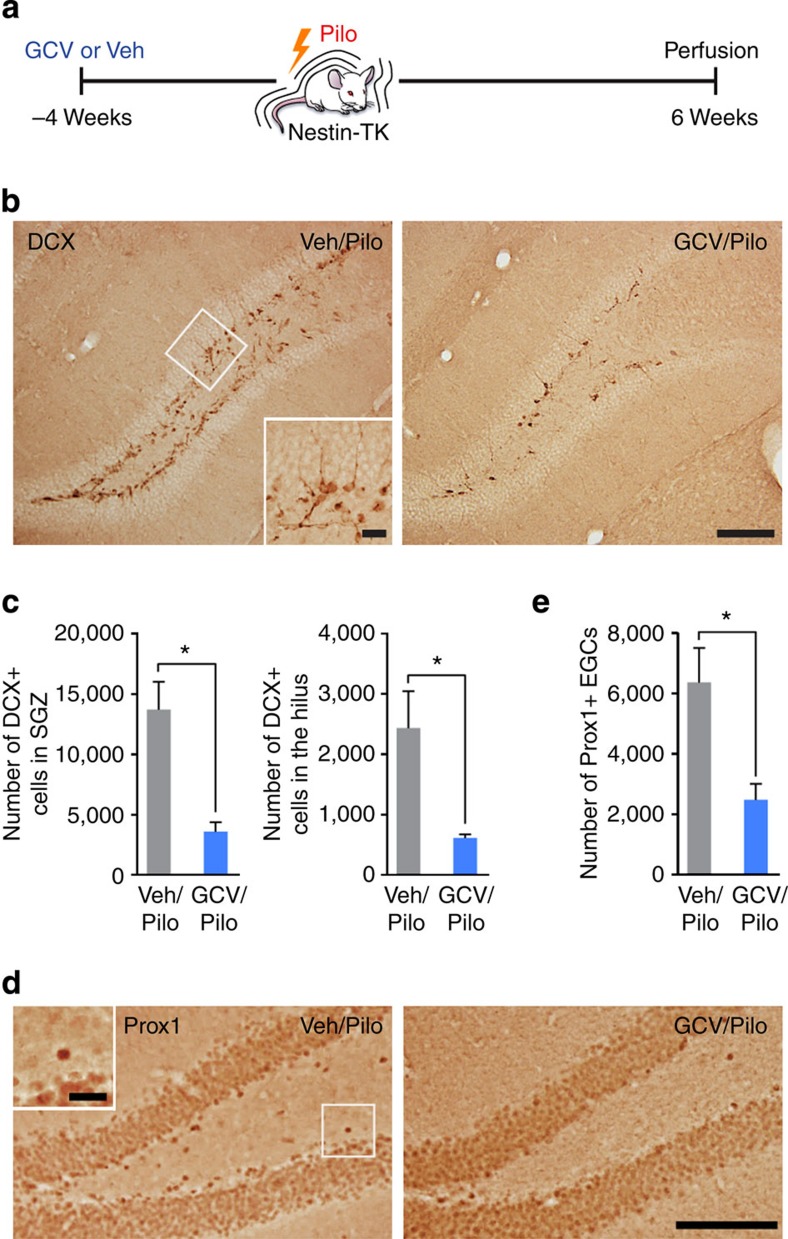
Ablating adult neurogenesis reduces aberrant neurogenesis. (**a**) Time line for histologic analysis. (**b**) Representative microscopic images from three independent experiments showing DCX immunoreactivity at 6 weeks after pilocarpine (Pilo) injection. Scale bar is 100 μm. Inset shows typical DCX-positive cells after pilocarpine. Scale bar is 20 μm. (**c**) A graph showing the number of DCX-expressing cells in the SGZ and the hilus in Veh/Pilo (*n*=8) and GCV/Pilo group (*n*=7). Student’s *t*-test, *P*=0.003, *t*(8.569)=4.156 for the left graph; Student’s *t*-test, *P*=0.020, *t*(7.137)=2.971 for the right graph. (**d**) Representative microscopic images from three independent experiments showing the dentate gyrus stained with Prox1, a marker for granule neurons. Scale bar, 100 μm. Inset shows a typical ectopic granule neuron. Scale bar, 20 μm. (**e**) A graph showing the number of EGCs in the hilus of Veh/Pilo (*n*=8) and GCV/Pilo group (*n*=8). Student’s *t*-test, *P*=0.012, *t*(9.871)=3.056. Data presented as mean±s.e.m. *P<0.05.

We next asked whether the reduced seizure frequency after ablation of adult neurogenesis in our study was due to changes in adult-generated neurons, such as the formation of EGCs and MFS. First, we assessed the number of DCX-expressing neuroblasts and immature neurons in the subgranular dentate gyrus and in the dentate hilus of Veh- or GCV-treated Nestin-TK mice. At 6 weeks after pilocarpine injection, when SRS were observed in all animals, animals that had ablation of neurogenesis by GCV administration (GCV/Pilo) had fewer DCX-expressing cells in the subgranular dentate gyrus and the hilus, compared with the group with no ablation of neurogenesis (Veh/Pilo; Fig. 5a–c). Furthermore, we found that Prox1-expressing hilar EGCs were significantly decreased in the GCV/Pilo group (Fig. 5d,e). However, MFS stained by zinc transporter-3 did not significantly differ between the Veh/Pilo and GCV/Pilo groups (Supplementary Fig. 2).

To begin to gain insight into important molecular regulators of aberrant neurogenesis, we found that NeuroD-expressing cells were increased in the dentate gyrus and the hilus after kainic acid (KA) induced seizures, compared with sham (Supplementary Fig. 3a–c). Next we asked whether NeuroD is required for the formation of seizure-induced neurons. To accomplish this, we utilized *Nestin-CreER*^*T2*^*;NeuroD*^*loxP/loxP*^;*Rosa26(R26R)-YFP* (cKO) mice and *Nestin-CreER*^*T2*^;*NeuroD*^*+/+*^;*R26R-YFP* (wild-type, WT) mice to delete NeuroD in nestin-expressing stem cells and their progeny after tamoxifen injection^31^. Three weeks following KA injection, we found that proliferating neuroblasts identified by triple labelling with YFP/DCX/Ki67 were decreased in NeuroD cKO mice, compared with WT littermates (Supplementary Fig. 3d–f). Moreover, NeuroD deletion before acute seizures led to the reduction of Prox1-positive granule cells in the granule cell layer and the hilus (Supplementary Fig. 3g,h), suggesting that targeting NeuroD can be an alternative approach of eliminating adult neurogenesis. Together our findings indicate that ablation of adult neurogenesis using two distinct approaches can decrease seizure-induced aberrant neurogenesis.

### SRS persisted after near-complete ablation of neurogenesis

**Figure 6:**
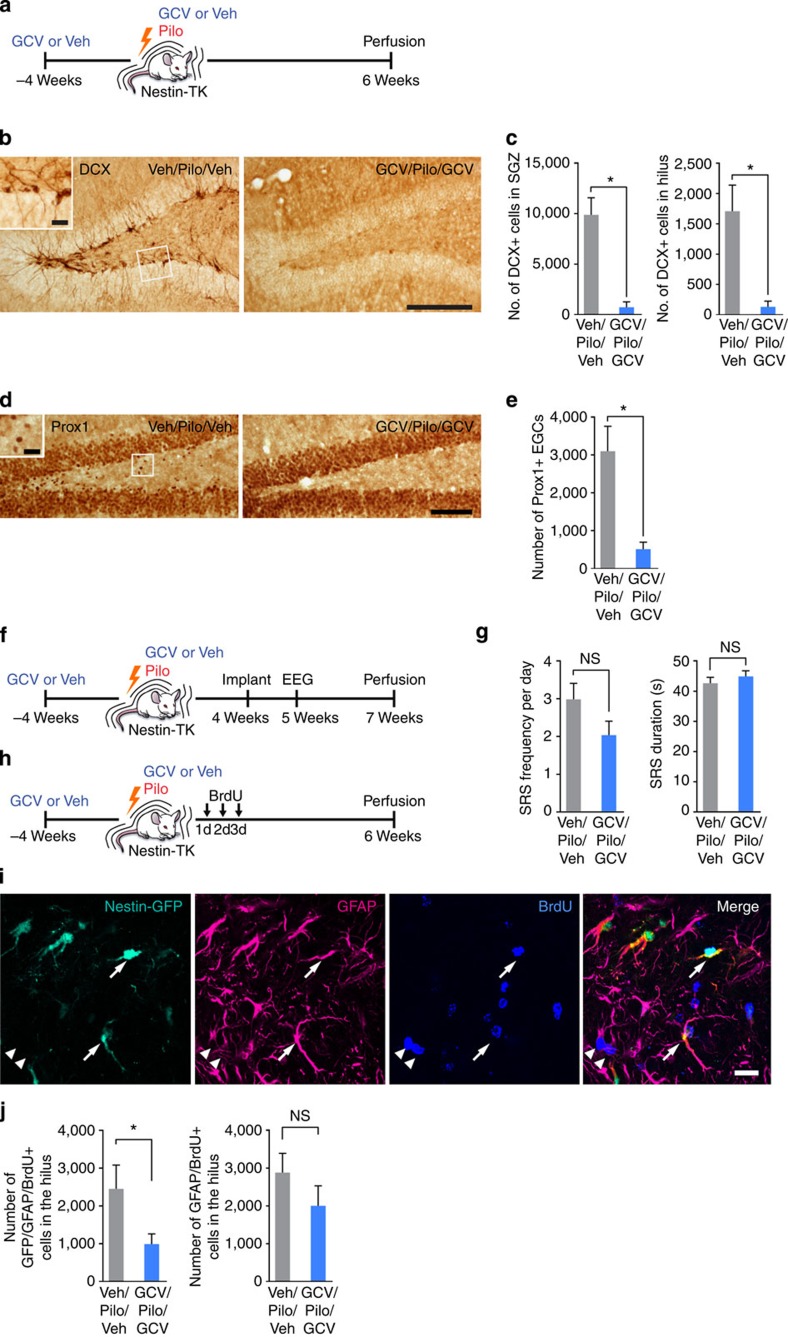
Seizures persist after near-complete ablation of neurogenesis. (**a**) Experimental design. (**b**) Microscopic images from three experiments showing DCX immunoreactivity at 6 weeks after pilocarpine (Pilo) injection. Scale bar, 200 μm. Inset shows typical DCX-positive cells in epilepsy. Scale bar, 20 μm. (**c**) Graphs showing the number of DCX-expressing cells in subgranular zone (SGZ) and the hilus in Veh/Pilo/Veh (*n*=11) and GCV/Pilo/GCV group (*n*=12). Mann–Whitney *U*-test, *P*<0.001, *U*=3.000 for the left graph; Mann–Whitney *U*-test, *P*<0.001, *U*=8.000 for the right graph. (**d**) Microscopic images from three experiments showing the dentate gyrus stained with Prox1, a marker for granule neurons. Scale bar, 100 μm. Inset shows typical EGCs in epilepsy. Scale bar, 20 μm. (**e**) A graph showing the number of EGCs in the hilus of Veh/Pilo/Veh (*n*=11) and GCV/Pilo/GCV group (*n*=12). Mann–Whitney *U*-test, *P*<0.001, *U*=8.000. (**f**) Experimental design. (**g**) Graphs showing SRS frequency and duration between Veh/Pilo/Veh (*n*=24) and GCV/Pilo/GCV groups (*n*=22). Mann–Whitney *U*-test, *P*=0.092, *U*=187.500 for the left graph; Student’s *t*-test, *P*=0.404, *t*(41)=−0.843 for the right graph. (**h**) Experimental design. (**i**) Confocal images from three experiments showing representative Nestin-TK GFP/GFAP/BrdU- (arrows) and GFAP/BrdU-immunoreactive cells (arrowheads) in the hilus. Scale bar, 20 μm. (**j**) Graphs showing the number of proliferating reactive astrocytes expressing Nestin-TK GFP, examined by Nestin-TK GFP/GFAP/BrdU+ cells and proliferating astrocytes not expressing Nestin-TK GFP, labelled as GFAP/BrdU staining, between Veh/Pilo/Veh (*n*=5) and GCV/Pilo/GCV group (*n*=6). Student’s *t*-test, *P*=0.049, *t*(9)=2.281 for the left graph; Student’s *t*-test, *P*=0.270, *t*(9)=1.174 for the right graph. Data presented as mean±s.e.m. *P<0.05. NS, not significant.

The reason why ablation of neurogenesis before acute seizures only reduced chronic seizure frequency, but did not completely prevent epilepsy, could be explained by neurons generated after seizures or other factors. To discriminate between these two possibilities, we treated Nestin-TK mice with GCV before and after pilocarpine to achieve near-complete ablation of neurogenesis (Fig. 6a). We found that after two rounds of GCV treatment (GCV/Pilo/GCV), DCX-expressing cells in the subgranular dentate gyrus and the hilus were fewer, compared with the group with no ablation of neurogenesis (Veh/Pilo/Veh; Fig. 6b,c). Furthermore, we found that Prox1-expressing hilar EGCs were significantly decreased in GCV/Pilo/GCV group (Fig. 6d,e). However, MFS stained by zinc transporter-3 still did not significantly differ between Veh/Pilo and GCV/Pilo groups, similar to ablation of neurogenesis before acute seizures (Supplementary Fig. 4). Unexpectedly, we found no significant difference in chronic seizures between the Veh/Pilo/Veh and GCV/Pilo/GCV groups, although there was a decreasing trend in the SRS frequency in the GCV/Pilo/GCV group (Fig. 6f,g).

As reactive astrocytes can express nestin^32^, we further examined if reactive astrocytes born after acute seizures could express Nestin-TK GFP and be ablated by the second round of GCV treatment (Fig. 6h). To test this hypothesis, we injected 5-bromo-2′-deoxyuridine (BrdU) after pilocarpine to label proliferating astrocytes and examined Nestin-TK GFP/GFAP/BrdU-expressing cells in the dentate gyrus. Indeed, at 6 weeks after pilocarpine, the number of Nestin-TK GFP/GFAP/BrdU-expressing cells in the hilus was significantly reduced in the GCV/Pilo/GCV group, whereas proliferating astrocytes not expressing Nestin-TK GFP (GFAP/BrdU-expressing cells) were not altered by GCV treatment after pilocarpine injection (Fig. 6i,j). These data indicate that GCV treatment after acute seizures can ablate a subpopulation of reactive astrocytes expressing Nestin-TK GFP, in addition to seizure-born neurons. As a result, the reason why suppressive effects on SRS were no longer observed after two rounds of GCV treatment could be due to the inhibition of seizures by ablating neurogenesis by one round of GCV and pro-excitatory effects of ablating astrocytes by the second round of GCV.

### Ablating neurogenesis rescues cognitive decline in epilepsy

**Figure 7 f7:**
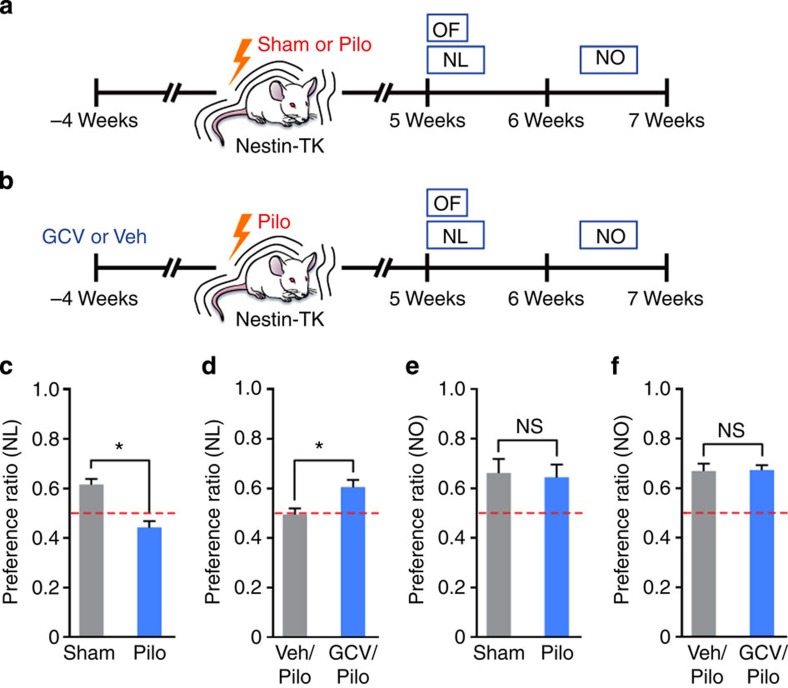
Ablating neurogenesis rescues cognitive decline in epilepsy. (**a**,**b**) Experimental time line is shown. (**c**) A graph showing the preference ratio of NO location (NL) test in sham mice (*n*=10) and pilocarpine-injected mice (*n*=9). Student’s *t*-test, *P*=0.0001, *t*(17)=5.017. (**d**) A graph showing the preference ratio of NL test between Veh (*n*=16) and GCV (*n*=17) groups. Student’s *t*-test, *P*=0.008, *t*(31)=−2.850. (**e**,**f**) Graphs showing the preference ratio in NO test (*n*=4 for sham, *n*=5 for Pilo; *n*=18 for Veh/Pilo, *n*=15 for GCV/Pilo). (**e**) Mann–Whitney *U*-test, *P*=1.000, *U*=10.000. (**f**) Student’s *t*-test, *P*=0.937, *t*(31)=−0.080. Data presented as mean±s.e.m. *P<0.05. NS, not significant.

In addition to the contribution of aberrant hippocampal neurogenesis to the formation of spontaneous seizures, abnormal neurons may also impair learning and memory, which is one of the most pervasive of epilepsy-related comorbidities^33^. We subjected Nestin-TK mice to a novel location (NL) recognition test, a hippocampus-dependent memory task^34^. Compared with the sham group, epileptic mice (Pilo) showed no preference for the NL, indicating impaired hippocampus-dependent memory function in chronic epilepsy (Fig. 7a,c). As expected, Veh/Pilo-treated mice showed a similar preference ratio as the Pilo group, whereas GCV/Pilo-treated Nestin-TK mice recognized the NL of an object, at a level similar to the sham group (Fig. 7b,d).

In the NO recognition task, a hippocampus-independent memory test^35^, all groups showed a preference for the new object relative to the familiar one (Fig. 7e,f), consistent with the idea that the rescued cognitive function observed in the NL test is related to the reduction of aberrant new neurons in the hippocampus. Importantly, we confirmed similar levels of locomotor activity in the open-field test between the groups (Supplementary Fig. 5). Taken together, these data indicate that ablation of neurogenesis in the pilocarpine model of epilepsy rescues hippocampal spatial memory impairment associated with chronic seizures.

### Ablating neurogenesis leads to long-term suppression of SRS

**Figure 8 f8:**
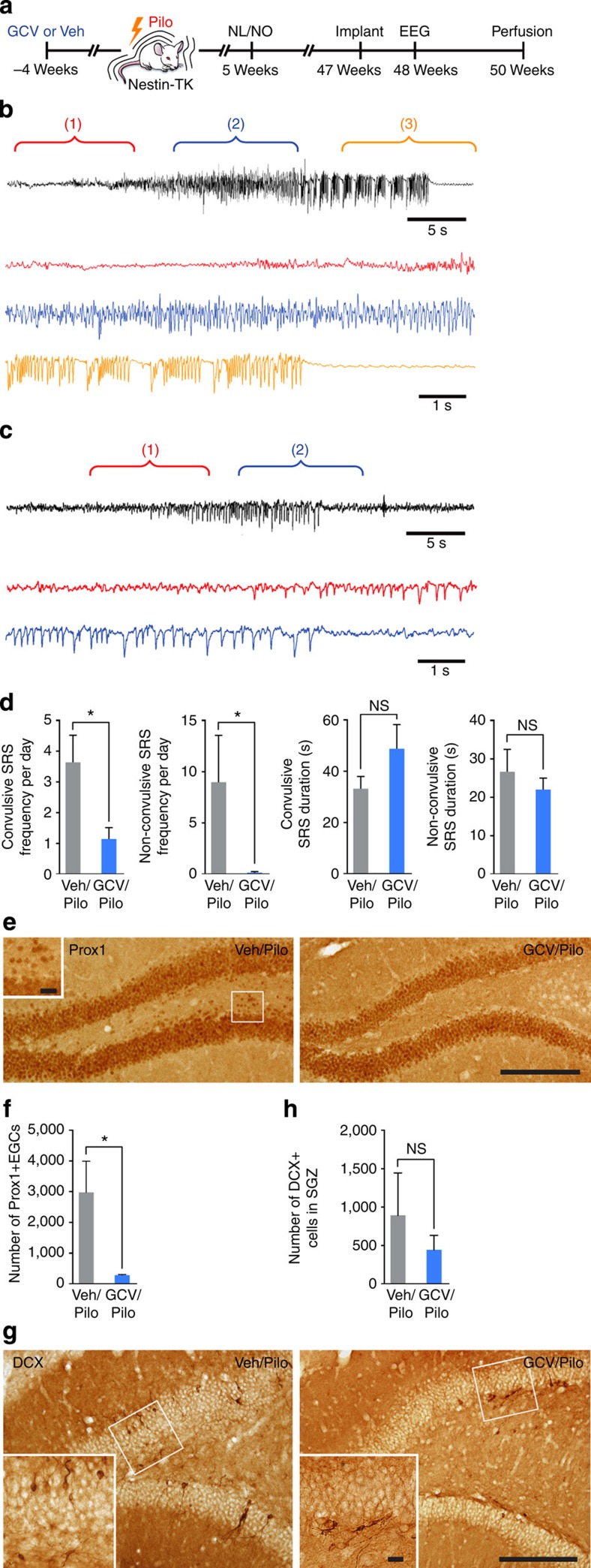
Ablating neurogenesis leads to long-term suppression of SRS. (**a**) Time line to show experimental design. (**b**) A representative EEG trace showing convulsive seizure activity from three independent experiments. Details are presented as initial (1), middle (2) and end sections (3). (**c**) A representative EEG trace showing non-convulsive seizure activity from three independent experiments. Details are presented as initial (1) and end sections (2). (**d**) Graphs showing the frequency and duration of convulsive SRS and non-convulsive SRS between Veh (*n*=8) and GCV (*n*=8) groups. Student’s *t*-test, *P*=0.028, *t*(9.396)=2.603; Mann–Whitney *U*-test, *P*=0.038, *U*=13.500; Mann–Whitney *U*-test, *P*=0.053, *U*=9.000; Mann–Whitney *U*-test, *P*=0.739, *U*=5.000 from left to right. (**e**) Microscopic images from three independent experiments showing the dentate gyrus stained with Prox1, a marker for granule neurons. Scale bar, 200 μm. Inset shows typical ectopic granule neurons. Scale bar, 20 μm. (**f**) A graph showing the number of EGCs in the hilus in Veh/Pilo (*n*=6) and GCV/Pilo group (*n*=4). Student’s *t*-test, *P*=0.045, *t*(5.004)=2.665. (**g**) Microscopic images from three independent experiments showing DCX immunoreactivity in epilepsy. Scale bar, 200 μm. Inset shows typical DCX-positive cells in SGZ. Scale bar, 20 μm. (**h**) A graph showing the number of DCX-expressing cells in the subgranular zone (SGZ) between Veh/Pilo (*n*=6) and GCV/Pilo (*n*=4) groups. Mann–Whitney *U*-test, *P*=0.831, *U*=11.000. Data presented as mean±s.e.m. *P<0.05. NS, not significant.

To examine whether ablated neurogenesis has a persistent effect on SRS frequency, we allowed the NL/NO-tested mice to age for ~1 year after pilocarpine injection and performed EEG monitoring for 2 weeks (Fig. 8a). To minimize stress-associated mortality in aged mice, we implanted epidural cortical screws and used a wireless EEG monitoring system instead of hippocampal depth electrodes and a tethered system used previously for young animals. Since there was only one channel to monitor recurrent seizures, we classified SRS as either convulsive (Fig. 8b, Supplementary Video 1) or non-convulsive (Fig. 8c, Supplementary Video 2) with video confirmation. Interestingly, the frequency of both convulsive and non-convulsive SRS was significantly lower in GCV-treated mice than Veh-treated animals, with no difference in SRS duration for each seizure (Fig. 8d), consistent with our data of young animals. Moreover, the number of hilar EGCs was markedly decreased by ablated neurogenesis (Fig. 8e,f), whereas the subgranular neurogenesis examined by DCX immunohistochemistry was comparable between Veh and GCV groups (although both groups showed reduced number and altered morphology of DCX cells characteristic of aged mice^36^,^37^), suggesting the recovery of subgranular neurogenesis after a single round of GCV-induced ablation of neurogenesis (Fig. 8g,h). Taken together, ablation of aberrant neurogenesis before acute seizures can permanently reduce the number of chronic seizures, thus reinforcing the therapeutic relevance of aberrant neurogenesis as a target for epilepsy.

## Discussion

Defining the cellular and molecular changes after an initial seizure episode responsible for the formation of chronic seizures is paramount for the development of effective anti-epileptogenic strategies. Our data show that blocking adult neurogenesis before acute seizures reduced SRS, whereas the ablation of neurogenesis pre- and post-acute seizures did not decrease the frequency of recurrent seizures. However, we also found that two rounds of GCV treatment killed proliferating reactive astrocytes together with seizure-generated granule neurons, so the question regarding the role of post-seizure neurogenesis in epileptogenesis is still open. In addition, we showed that ablation of neurogenesis before acute seizures alleviated epilepsy-associated hippocampal memory deficits. We also found that NeuroD is required for seizure-generated neurons, although future work is needed to determine the functional consequences of deleting NeuroD in chronic seizures or associated cognitive function. Finally, a single ablation of adult neurogenesis showed a long-lasting effect on SRS by reducing seizure frequency, even at a late chronic stage of the disease, further emphasizing the contributory role of aberrant neurogenesis in epileptogenesis.

A major goal in developing specific interventions for intractable epilepsy is to identify the cellular culprit(s) that contribute to neural circuits underlying seizure formation. We found fewer hilar EGCs as a result of ablating neural progenitors, which correlated with the reduction of SRS frequency in both young and aged animals. Hilar EGCs receive more excitatory inputs from mossy fibres than cells in the granule cell layer^23^,^38^. Thus, hilar EGCs can create more excitatory circuitry in the dentate gyrus and CA3 subregion of the hippocampus and function as hyperexcitable hubs, causing recurrent seizures^39^. Computational work further supported this idea, showing that the addition of a few granule cell hubs remarkably increased the excitability of the network and lowered the threshold for seizure initiation^20^,^40^, possibly leading to increased SRS frequency. We thus speculate that the reduction of hilar EGCs in the network through the ablation of neurogenesis could attenuate the formation of aberrant excitatory circuits in the hippocampus, and increase the threshold for seizure initiation, thereby decreasing SRS frequency. Indeed, a less-specific approach showed that decreasing hilar EGCs decreases seizure frequency in an earlier study^13^. However, it is possible that other features of seizure-induced aberrant neurogenesis including hilar basal dendrites and cellular hypertrophy may also contribute to the development of epilepsy.

Another common pathological finding in TLE is MFS^10^,^17^,^18^. Prior reports indicate that only adult-generated granule neurons develop MFS^41^,^42^; thus, it is surprising that we did not observe a difference in MFS after the ablation of neurogenesis. It is possible that non-ablated neurons (either still dividing or post mitotic at the time of pilocarpine) after 4 weeks of GCV treatment can undergo changes in MFS, possibly compensating for the loss of MFS from ablated adult-generated neurons. Alternatively, seizure-born neurons may increase MFS only in the GCV group, perhaps in response to ablation of neurogenesis. However, regardless of the detailed cell type(s) that contribute to MFS, our results showing no change in MFS while SRS is reduced, support the notion that MFS does not influence chronic seizures^12^,^18^.

It is surprising that we did not observe a reduction of chronic seizures after near-complete ablation of neurogenesis, which is in contrast to the single round of GCV treatment before acute seizures. We speculate that there could be two possible reasons. First, two rounds of GCV treatment not only decreased aberrant neurogenesis but also deleted a population of proliferating astrocytes, due to seizure-induced expression of Nestin-TK in reactive astrocytes. Since astrocytes have been shown to influence excitability greatly, removing this population could potentially result in unexpected changes in SRS. Second, a population of newborn neurons that can suppress chronic seizures was ablated by two rounds of GCV treatment. Consistent with the first possibility, we found two rounds of GCV treatment ablated seizure-generated neurons and proliferating reactive astrocytes. Considering the uncertain role of astrocytes in epilepsy development^43^, ablation of reactive astrocytes might explain why the suppressing effect of ablated neurogenesis on chronic seizures was not detected. Clearly, better tools are required to dissect the sole contribution of seizure-generated neurons in epileptogenesis.

Another possibility to consider is that adult-generated neurons after seizures may be heterogeneous^44^. Seizure activity stimulates the generation of adult-generated neurons migrating into the granule cell layer as well as ectopically into the hilus. Moreover, even in the granule cell layer, seizure-born neurons are morphologically heterogeneous in terms of spine density and number^44^, possibly contributing to a mixture of hyper- and hypoexcitable cells. Consistent with the idea that functionally heterogeneous populations of adult-born neurons exist and new neurons may also be hypoexcitable, emerging data indicate that newborn neurons may serve as an inhibitory gate to prevent the propagation of seizure activity entering the hippocampus^45^. Furthermore, we cannot assume that EGCs are always pathological, as they can be found in small numbers in the normal hippocampus^20^,^46^,^47^,^48^. Thus, a future challenge will be to identify unique tools that distinguish between aberrant and normal (or beneficial) adult-generated neurons.

We also show that the ablation of neurogenesis improves epilepsy-associated cognitive decline. Cognitive decline observed in TLE patients includes problems with memory, executive function and low levels of intelligence^49^. Among them, memory impairment is the most common problem, which is not surprising considering that the temporal lobe is required for memory formation^49^. Because epileptic seizures may produce adult-generated neurons that contribute to the formation of aberrant hippocampal circuits and disrupt the normal network^22^,^38^,^50^, seizure-induced abnormal granule cells may cause epilepsy-associated learning and memory deficits. Consistent with this idea, we previously discovered that histone deacetylase inhibitors such as VPA potently suppressed seizure-induced neurogenesis and restored hippocampal-dependent memory function^27^. Since VPA is reported to have additional targets^51^, we further confirmed this hypothesis using a genetic ablation model to address the role of aberrant neurogenesis in epilepsy-associated memory decline. Ablation of adult neurogenesis before acute seizures successfully normalized hippocampal-dependent memory deficit observed in non-ablated epileptic mice. Currently, we lack specific tools to completely dissociate the contribution of aberrant new neurons—from the seizures themselves—to spatial memory dysfunction. Thus, although both groups experienced fewer than three SRS per day (on average) in the pilocarpine model, it is possible that spatial memories may still be disrupted. However, we believe that this is the first report to demonstrate the links between seizure-induced aberrant hippocampal neurogenesis and cognitive decline associated with epilepsy.

Since epileptogenesis is a complex process involving multiple brain regions and cell types, it is essential to identify which brain regions and circuit elements are critically involved in seizure generation. We showed that the ablation of aberrant hippocampal neurogenesis only reduced SRS frequency, but did not prevent the occurrence of spontaneous seizures. Since the pilocarpine model affects not only the hippocampus, but also extrahippocampal regions such as the piriform and entorhinal cortices, amygdala and thalamus^52^, it is possible that the remaining seizures still observed after ablation of neurogenesis might be chronic seizures involving extrahippocampal regions. However, current animal models of epilepsy such as intrahippocampal KA injection are not ideal to address this question, since there is destruction of the neurogenic niche in the injected hippocampus^53^,^54^. Thus, it will be interesting to examine whether aberrant neurogenesis can prevent the occurrence of hippocampus-associated chronic seizures with a model that can specifically manipulate hippocampal circuitry without affecting other brain regions in the future. In this manner, we may be able to address the contribution of new dentate neurons to the initiation of seizures. A future goal is to confirm our work in a hippocampus-originated model of chronic epilepsy where the ability to promote neurogenesis is not compromised.

Finally, as epilepsy is typically a life-long disease with debilitating recurrent seizures, it is crucial to develop long-lasting modalities to prevent, or at least alleviate, chronic seizures. To achieve this goal, two possible approaches are targeting the latent period before the establishment of epilepsy or the chronic stage of epilepsy when spontaneous seizures are already observed, hoping to permanently alter the natural course of epilepsy. In this paper, we provide evidence that blocking hippocampal neurogenesis before acute seizures redirects the epileptogenic course to persistently mitigate the frequency of spontaneous seizures even during aging. Since a single ablation of adult neurogenesis was sufficient to produce long-lasting suppressive effects on recurrent seizures at a late chronic stage of epilepsy, our finding can greatly strengthen the therapeutic impact for reducing seizures throughout life. Now with knowledge from our work that seizure-induced neurons contribute to recurrent seizures and associated memory impairment, we may be able to develop more effective treatment strategies for intractable epilepsy by targeting aberrant hippocampal neurogenesis. Our studies also provide a cautionary note regarding neuroregenerative approaches whereby induction of aberrant neurogenesis may exacerbate rather than mitigate disease symptoms.

## Methods

### Mice

All mice (C57BL/6 background, male and female) were bred and housed in the animal facility with a 12-h light, 12-h dark cycle with no more than five mice per cage, unless stated otherwise. Nestin-δ-HSV-thymidine kinase-EGFP transgenic (Nestin-TK) mice^30^ were genotyped by PCR using genomic DNA and primers for Nestin-TK (5′-GCC TTG ACC AGG GTG AGA TA-3′, 5′-ATG CTG CCC ATA AGG TAT CG-3′) and GFP (5′-GAG CTG GAC GGC GAC GTA AAC-3′, 5′-CGT TGT GGC TGT TGT TAG TTG TAC-3′). Nestin-TK mice were crossed with C57BL/6 obtained from Harlan Laboratories to generate transgenic and WT mice. Male and female mice at 6 weeks of age were inserted with an osmotic mini pump (Model 2004; Alzet) filled with either GCV (subcutaneously) at 150 mg kg^−1^ per day or distilled water, which was removed 4 weeks later. All the experiments were performed in compliance with the animal care guidelines issued by the National Institutes of Health and by the Institutional Animal Use and Care Committee at University of Texas Southwestern Medical Center.

### Chemoconvulsant models of TLE

Nestin-TK mice at 10 weeks of age were administered scopolamine methyl nitrate (intraperitoneally (i.p.); 2 mg kg^−1^; Sigma-Aldrich S2250) and terbutaline hemisulfate salt (i.p.; 2 mg kg^−1^; Sigma-Aldrich T2528) to block peripheral effects of pilocarpine and dilate the respiratory tract, respectively. Thirty min later, pilocarpine hydrochloride (i.p.; Sigma-Aldrich P6503) at 245 mg kg^−1^ for males and 270 mg kg^−1^ for females was injected, and mice were placed in an incubator maintained at 31 °C (ThermoCare). Acute seizures were behaviourally monitored using a modified Racine’s scale^55^ (stage 1, mouth and facial movement; stage 2, head nodding; stage 3, forelimb clonus; stage 4, rearing with forelimb clonus; stage 5, rearing and falling with forelimb clonus). Once status epilepticus began (defined by continuous tonic clonic convulsive seizures), mice were placed at room temperature for 3 h and returned to the incubator after seizure activity was reduced with diazepam (10 mg kg^−1^; Sigma-Aldrich D0899). Only mice showing status epilepticus were selected for further processing and osmotic mini pumps were removed. Mice were administered 5% dextrose solution (i.p.; 1 ml) and saline (i.p.; 1 ml) to facilitate their recovery. At 2 days after pilocarpine injection, mice were returned to their cages and randomly assigned to the experiments for histology, video/EEG and behavioural tests. One cohort of animals were injected with BrdU (i.p.; 150 mg kg^−1^; Sigma-Aldrich B5002) once a day on days 1–3 after pilocarpine to label proliferating cells after acute seizures.

### Immunohistochemistry

Mice were anaesthetized and perfused transcardially with cold 4% paraformaldehyde (PFA) in 0.1 M PBS. Brains were removed and postfixed in 4% PFA overnight, then cryoprotected in 30% sucrose in 0.1 M PBS. Brains were bisected and half-brains were coronally sectioned 30 μm thick on a freezing microtome. Immunohistochemistry was performed with either tissue mounted on charged slides or free-floating tissue sections. Slides underwent antigen retrieval using 0.01 M citric acid, pH 6.0 at 100 °C for 15 min, followed by 12 min in TBS at room temperature. Staining with free-floating tissue sections was the same except for the antigen retrieval step that was omitted. For Tyramide-Plus signal amplification^31^, we removed endogenous peroxidase activity by incubating sections with 0.3% H_2_O_2_ for 30 min at room temperature. Nonspecific binding was blocked with 3% normal donkey serum and 0.3% Triton-X-100 in TBS for 1 h at room temperature. Primary antibodies in this study were chosen based on the validation results by the manufacturer: goat anti-NeuroD (1:1,000, Santa Cruz Biotechnology sc-1084), chicken anti-GFP (1:8,000, Aves Lab GFP-1020), goat anti-DCX (1:2,000 for free-floating sections, 1:500 for tissue mounted on slides, Santa Cruz Biotechnology sc-8066), guinea pig anti-DCX (1:2,000, Millipore AB2253), rabbit-anti-Prox1 (1:500, Millipore AB5475), mouse anti-GFAP (1:1,000, Millipore MAB360), rat-anti-BrdU (1:500, Accurate Chemical & Scientific Corporation OBT0030G). For double or triple labelling, primary antibodies were simultaneously incubated (for example, NeuroD/DCX, GFP/GFAP) and further processed for each antibody. For NeuroD and GFAP, a fluorescent-tagged secondary antibody was used (1:300, Jackson ImmunoResearch). For GFP and DCX, primary antibody incubation was followed with an appropriate biotin-tagged secondary antibody (1:200, Jackson ImmunoResearch) for 1 h at room temperature followed by ABC (Vector Laboratories PK-6100) for 1 h and Tyramide-Plus signal amplification (1:50, PerkinElmer NEL701001KT) for 10 min. Sections were counterstained with DAPI (4',6-diamidino-2-phenylindole; 1:5,000, Roche 236276). For DCX and Prox1, after biotin-tagged secondary antibody followed by ABC labelling was completed, sections were visualized with metal-enhanced DAB substrate kit (Thermo Scientific 34065). For BrdU triple staining, after GFP and GFAP staining is finished, the sections were mounted and further fixed with PFA for 30 min. Then, the slides were permeabilized with 0.1% trypsin in 0.1 M Tris and 0.1% CaCl_2_ for 10 min, followed by denaturation with 2 N HCl solution for 25 min. Slides were then blocked and processed as described above. For Nissl staining, tissues were mounted on slides and underwent a series of hydration steps from 100% EtOH to tap water (100, 95, 90, 80 and 70% EtOH for 3 min each) and were then incubated with 0.1% cresyl violet solution (Sigma-Aldrich C5042) for 15 min. Excessive staining was removed by 95% EtOH with 0.1% glacial acetic acid, and the slides were dehydrated with solutions of 100% EtOH, 50% EtOH/xylene, and 100% xylene. For FJC staining (Histo-Chem Inc.), tissues mounted on the gelatin-coated slides were hydrated from 100% EtOH, 70% EtOH to water for 1 min each and were incubated with 0.06% potassium permanganate solution for 14 min. After rinsing with water for 1 min, the sections were incubated in 0.001% FJC solution for 30 min. Slides were dried overnight, followed by incubation with 100% xylene for 3 min.

### Microscopic analysis and quantification

Quantification of cell number in the hippocampus was performed using either an upright microscope (BX60; Olympus) or a confocal microscope (LSM510; Carl Zeiss Microscopy) by an observer blinded to experimental groups. Subgranular and hilar zones were defined as the area within and beyond the diameter of one granule cell from the margin of granule cell layer, respectively. Immunoreactive cells were quantified in every twelfth 30-μm coronal section throughout the dentate gyrus. For double- or triple-stained sections, confocal images in *Z* planes were scanned to quantify marker-positive cells. The numbers counted from each section were added and multiplied by 24 to estimate the total number of cells in one animal.

### Video/EEG monitoring

Video/EEG recording was performed 5–7 weeks after pilocarpine injection or during acute pilocarpine-induced status epilepticus. One week before EEG recording, mice were stereotaxically implanted with recording electrodes under gas anaesthesia with ~1–2% isoflurane in a 1 l min^−1^ mixture of 70% nitrous oxide and 30% oxygen. Two epidural electrodes made from #00-90 × 1/8 inch stainless steel screws were placed at coordinates from bregma: A–P −0.5 mm, lateral −1.5 mm (left frontal) and A–P −3.5 mm lateral 2.0 mm (right occipital). In addition, bilateral depth electrodes made from 125 μm diameter stainless steel were implanted into the dorsal hippocampus at coordinates from bregma: A–P −2.5 mm±2.5 mm to a depth of 2.5 mm. Reference and ground screw electrodes were placed over the olfactory bulb and cerebellum, respectively. Electrodes were attached by a flexible wire (Kynar, 30 gauge) to a custom 6-pin micro connector (Omnetics) and secured with dental acrylic. Mice received the analgesic Buprenorphine (subcutaneously; 0.05 mg kg^−1^) as necessary following surgery and were allowed to recover for at least 7 days before EEG recording. Mice surviving implant surgery were individually placed in a custom acrylic recording cage (Marsh Designs) and connected to a Tucker-Davis Technologies RZ2/PZ3 neurophysiology workstation through a flexible cable suspended from the top of the cage with an interposed commutator to allow mice free access to food and water without twisting the cable. Continuous video/EEG (300 Hz sampling) was recorded for each mouse simultaneously for up to 14 days and read off-line (LabChart Reader; ADInstruments) by a user blinded to the experimental grouping for the presence of seizures and epileptiform activity. Generalized seizures were defined by repetitive epileptiform spiking activity (≥3 Hz) that persisted in all electrodes for >3 s. Seizure activity was marked at the beginning and end of each event to account for seizure duration the number of seizures for each mouse was noted. After EEG recording was completed, mice were killed and the location of hippocampal depth electrodes was confirmed with Nissl staining. For aged mice, we used a wireless EEG monitoring system (Data Sciences International) to minimize the stress-associated mortality during EEG recording. One week before EEG recording, mice were implanted with epidural electrodes placed at coordinates from bregma: A–P −2.0 mm, lateral 2.2 mm and A–P 1.0 mm, lateral 2.0 mm. Cortical electrodes were connected to wireless EEG transmitters (TA11ETAF10; Data Sciences International) positioned in the subcutaneous pocket created in the back. Video-EEG data, collected 24/7 for 2 weeks, were analysed using Neuroscore software (version 3.0; Data Sciences International) by a user blinded to experimental groups. Convulsive seizures were defined by a burst of spiking activity (≥3 Hz) that persisted for >3 s with high amplitudes (>2 × background activity) and tonic clonic behaviours confirmed by video, whereas non-convulsive seizures were defined by the same EEG criteria but without behavioral seizure activity greater than stage 2 from a modified Racine’s scale.

### Behavioural tests

Behavioural tests were conducted from 5–7 weeks after pilocarpine or saline injection, starting with open-field test followed by NL and NO recognition tasks. Locomotor activity in the open-field box was assessed during the 15 min habituation phase without objects in the NL task (Day 1). Mice were positioned in the center of the box, and then individual total distance moved during the first 10 min of habituation was automatically recorded using video tracking system (Noldus Information Technology). As for NL and NO tests, which are based on the spontaneous preference of rodents for novelty and their ability to remember previously encountered location and objects, NL and NO tasks are hippocampal-dependent^56^,^57^ or -independent memory^58^, respectively. In this study, we modified the published methods^34^,^56^ to adapt these tasks to seizure-prone mice. Each task was comprised of three phases (habituation, familiarization and testing) on separate days. All phases were performed in a blue-squared open-field box (44 × 44 × 30 cm) under dim light condition (60 lux) between 5:00 and 8:00 hours. All mice tested were acclimated in the testing room using a red light at 18:00 hours on the day before each phase. In the NL task, each mouse was habituated in the open-field box without objects for 15 min (Day 1) and the following day with two identical objects (50 ml tubes filled with water, object A) for 15 min (Day 2). In the familiarization phase for NL task (Day 3), mice were subjected to a 1-min rehabituation to the empty box and then placed in a holding cage. Immediately after two identical objects (black clips, object B) were placed in diagonal near the corners in the box (~5 cm from the walls), mice were returned to the box for familiarization. The mice were allowed to freely explore until they accumulated a total of 30 s exploring both objects. Exploration was defined as the mouse contacting the object with its whiskers, nose, or front paws. We ensured that every mouse spent the same amount of time exploring the objects and avoided any bias due to differences in individual levels of exploration by removing the animal from the box once it had explored the objects for a total of 30 s. Also, behaviours such as sitting on object, looking around or resting against object were not counted as exploratory time. Once reaching the 30 s criteria, mice were then removed from the box and returned to their home cages. The mice that did not reach the criteria within 15 min were excluded from further testing. In the NL testing phase (Day 4), mice were subjected to a 1-min rehabituation to the empty box and then placed in a holding cage. Immediately after two identical objects (object B), one of which was moved to a NL, were placed near the corners in the box, mice were reintroduced into the box, and their exploratory behaviours were recorded by video camera for 10 min, and analysed later. Time spent exploring each object for 10 min was scored. In the NO task, each mouse was habituated in the open-field box with two identical objects (object A) for 15 min (Day 8). In the familiarization phase for NO task (Day 9), mice were subjected to a 1-min rehabituation to the empty box and then placed in a holding cage. Just after two identical objects (metal cones, object C) were placed near the corners on one wall in the box (~5 cm from the walls), mice were returned to the box for familiarization, allowed to freely explore until reaching the 30 s criteria, and then removed from the box and returned to their home cages. In the NO testing phase (Day 10), mice were subjected to a 1-min rehabituation to the empty box and then placed in a holding cage. Immediately after two objects, one of which was a novel one (plastic doll, object D) and the other was a familiar one (object C), were placed in the same positions as during familiarization, mice were reintroduced into the box, and their exploratory behaviours were recorded by video camera for 10 min, and scored later. Location of the moved object in the NL and position of the object used as novel or familiar in the NO were counterbalanced across mice. To analyse the cognitive performance, preference of NL or object was calculated as the time spent exploring the object in the NL or NO divided by the cumulative time spent exploring both objects. The open-field box was cleaned thoroughly with disinfectant solution after each trial to ensure the absence of olfactory cues.

### Statistics

All of the data are expressed as mean±s.e.m. Experimental groups were assigned by simple randomization. No statistical methods were used to predetermine sample sizes, but our sample sizes are similar to those reported in previous publications^12^,^17^,^56^,^57^. Data that passed our selection criteria were collected blind. Once data were generated, all were included for analysis except for the NL test (Fig. 7d), where one mouse in Veh/Pilo group showed an extremely low preference ratio (0.021) and was thus removed from the dataset. SPSS (version 21.0, IBM SPSS Corp.) software was used for statistical comparison. Statistical differences were analysed using two-tailed Student’s *t*-test for the data with equal variances (Figs 1e and 2f left, 2f right, 4c, 4e left, 6g right, 6j left, 6j right, 7c, 7d, 7f, [Fig f7], [Fig f7], [Fig f7] or Student’s *t*-test with Satterthwaite’s correction for the data with unequal variances (Fig. 4e right, 5c left, 5c right, 5e, 8d far left, 8f). If normal distribution was not assumed, Mann–Whitney *U*-test was performed (Figs 1c, 3e,f, h and 6c left, 6c right, 6e, 6g left, 7e, 8d left, 8d right, 8d far right, 8h), [Fig f8]. Values of *P*<0.05 were considered significant.

## Additional information

**How to cite this article:** Cho, K. *et al.* Aberrant hippocampal neurogenesis contributes to epilepsy and associated cognitive decline. *Nat. Commun.* 6:6606 doi: 10.1038/ncomms7606 (2015).

## Supplementary Material

Supplementary Information Supplementary Figures 1-5, Supplementary Methods and Supplementary References.

Supplementary Video 1 Representative convulsive spontaneous recurrent seizures. Real time video-EEG recording shows a mouse with electrographic seizures and convulsive behavior.

Supplementary Video 2 Representative non-convulsive spontaneous recurrent seizures. Real time video-EEG recording shows a mouse with electrographic seizures but no apparent behavioral changes.
