# Supplementary material for: Aberrant hippocampal neurogenesis contributes to epilepsy and associated cognitive decline
Source: Nat Commun. 2015 Mar 26;6:6606. doi: 10.1038/ncomms7606 (PMC4375780; doi:10.1038/ncomms7606)
Supplement: Supplementary Information — Supplementary Figures 1-5, Supplementary Methods and Supplementary References. [file ncomms7606-s1.pdf]

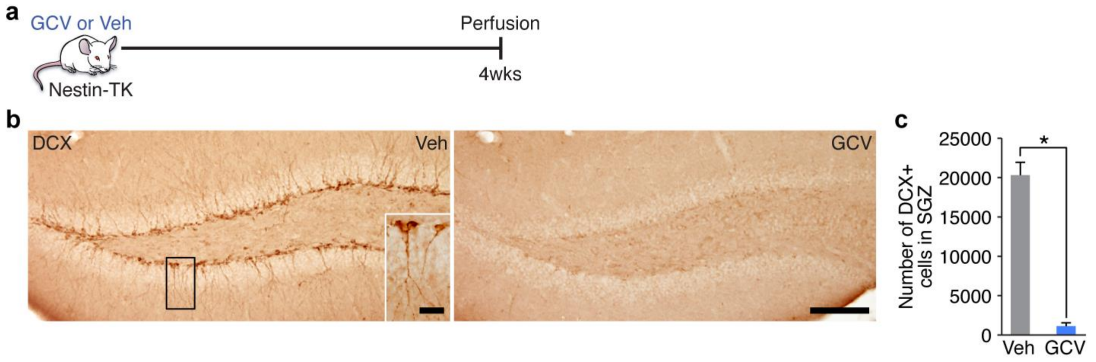

Supplementary Figure 1 | Genetic ablation of adult-born granule neurons (**a**) Time line showing the experimental design. (**b**) Representative microscopic images from three independent experiments showing dentate gyrus doublecortin (DCX) immunostaining in mice treated with either vehicle (Veh) or ganciclovir (GCV) for 4 weeks. Scale bar is 100 μm. Inset shows the cells expressing DCX. Scale bar is 20 μm. (**c**) A graph showing the number of DCX-expressing newborn neurons in the dentate gyrus in Veh (n=4) and GCV group (n = 5). Mann-Whitney U test, $P = 0.016$, $U < 0.001$. Data present as mean ± s.e.m. *$P < 0.05$. Nestin-TK, Nestin-δ-HSV-thymidine kinase-EGFP transgenic mice.

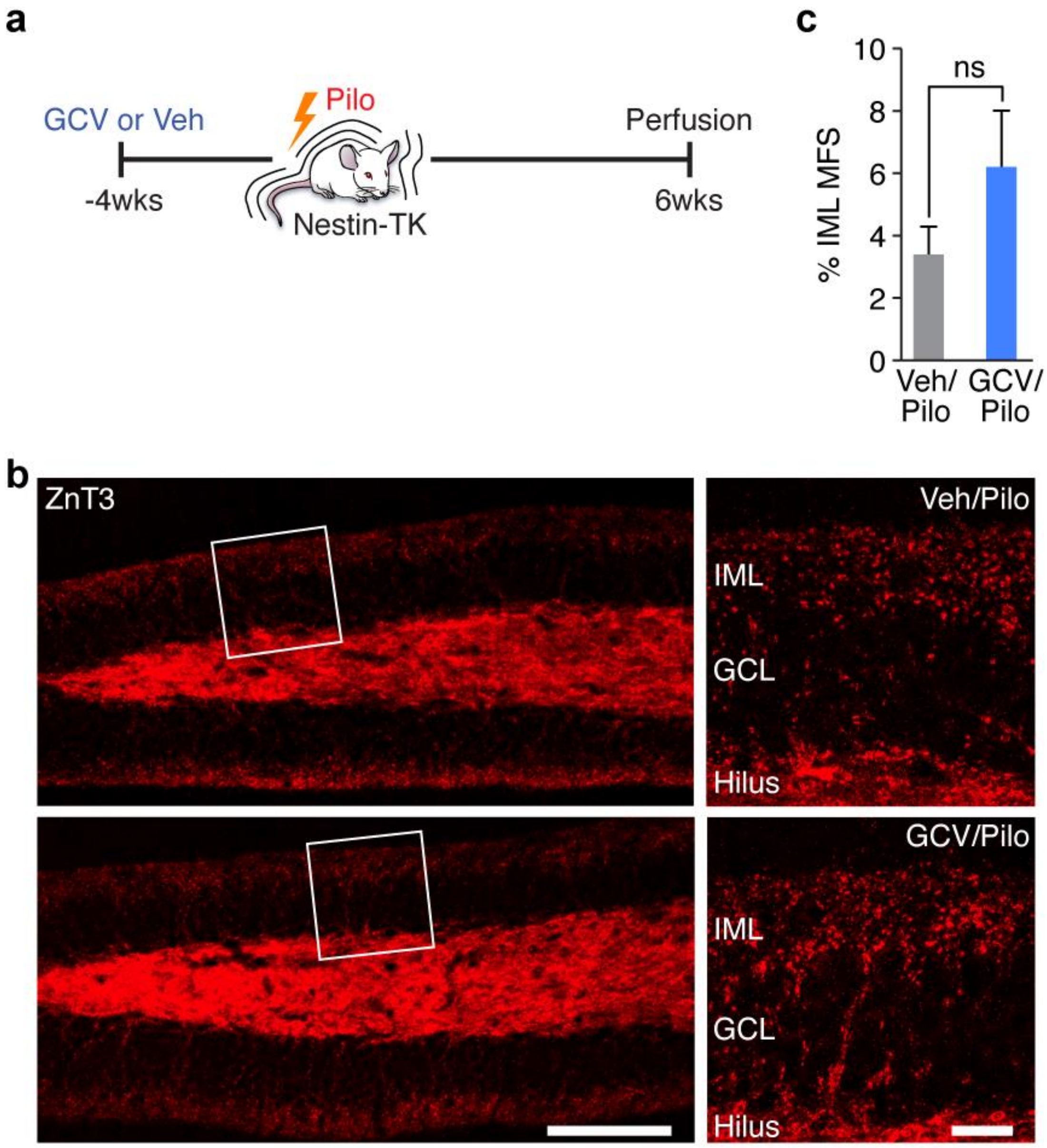

Supplementary Figure 2 | Ablating neurogenesis before seizures does not affect MFS (a) Time line to show experimental design. **(b)** Representative microscopic images from three independent experiments showing hippocampal zinc transporter-3 (ZnT3) immunoreactivity, a marker for the axons of granule cells, in epilepsy. Scale bar is 100 μm. Higher magnification images of ZnT3 immunoreactivity are shown in the right panel. Scale bar is 20 μm. **(c)** A graph measuring ZnT3-positive areas in IML of the dentate gyrus between Veh/Pilo (n = 8) and GCV/pilo groups (n = 7). Mann-Whitney U test, P = 0.298, U = 19.000. Data present as mean ± s.e.m. ns, not significant. GCV, ganciclovir; Veh, vehicle; Nestin-TK, Nestin-δ-HSV-thymidine kinase-EGFP transgenic mice; Pilo, pilocarpine; IML, inner molecular layer; GCL, granule cell layer; MFS, mossy fiber sprouting.

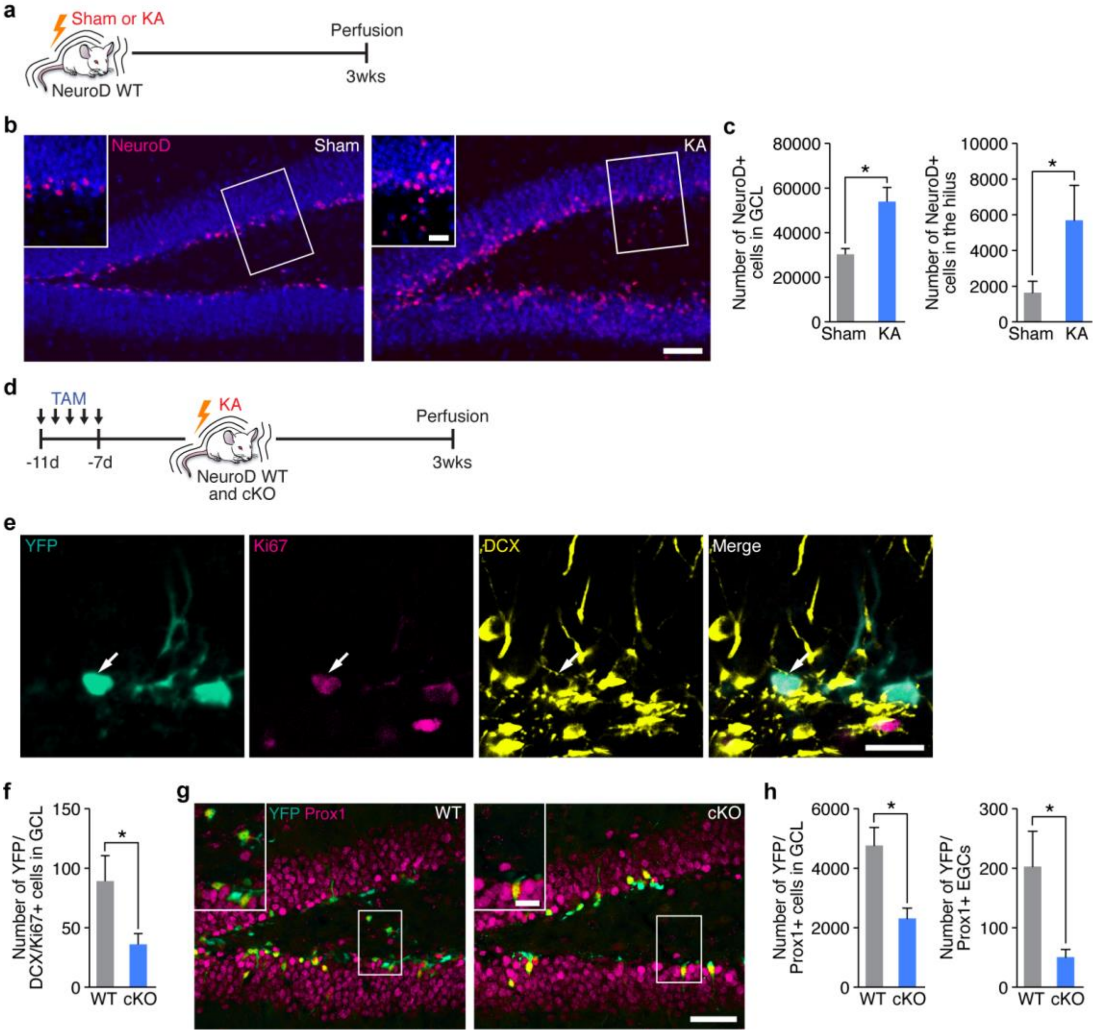

Supplementary Figure 3 | NeuroD is required for seizure-induced neurogenesis (**a**) Time line to show experimental design. (**b**) Representative fluorescent images from four independent experiments showing NeuroD induction in the dentate gyrus by kainic acid (KA). Scale bar is 50 µm. Insets show NeuroD-expressing cells in the dentate gyrus at a higher magnification. Scale bar is 20 µm. (**c**) Graphs showing NeuroD-expressing cells in the granule cell layer (GCL) and the hilus between sham and KA groups (n = 6 per group). Mann-Whitney U test, $P = 0.004$, U = 0.000 for the left graph; Mann-Whitney U test, $P = 0.045$, U = 5.500 for the right graph. (**d**) Experimental time line. Tamoxifen (TAM) was injected for 5 days to label newborn cells, followed by KA injection one week later. (**e**) Representative confocal images from three independent experiments showing a YFP-, Ki67-, and DCX-positive cell; a proliferating neuroblasts (arrow). Scale bar is 20 µm. (**f**) A graph showing the number of YFP/DCX/Ki67-expressing cells between NeuroD wild-type (WT) (n = 7) and conditional knockout (cKO) group (n = 8). Student's *t*-test, $P = 0.032$, $t(13) = 2.397$. (**g**) Representative confocal images from five independent experiments showing YFP/Prox1-immunoreactive cells in the dentate gyrus. Scale bar is 50 µm. Insets show YFP/Prox1-labeled cells in GCL and the hilus. Scale bar is 20 µm. (**h**) Graphs showing the number of YFP/Prox1-expressing cells in GCL and the hilus between NeuroD WT (n = 9) and cKO group (n = 10). Student's *t*-test, $P = 0.002$, $t(17) = 3.610$ for the left graph; Mann-Whitney U test, $P = 0.018$, U = 16.500 for the right graph. Data present as mean ± s.e.m. $^*P < 0.05$. YFP, yellow fluorescent protein; DCX, doublecortin; Prox1, prospero homeobox 1; EGCs, ectopic granule cells.

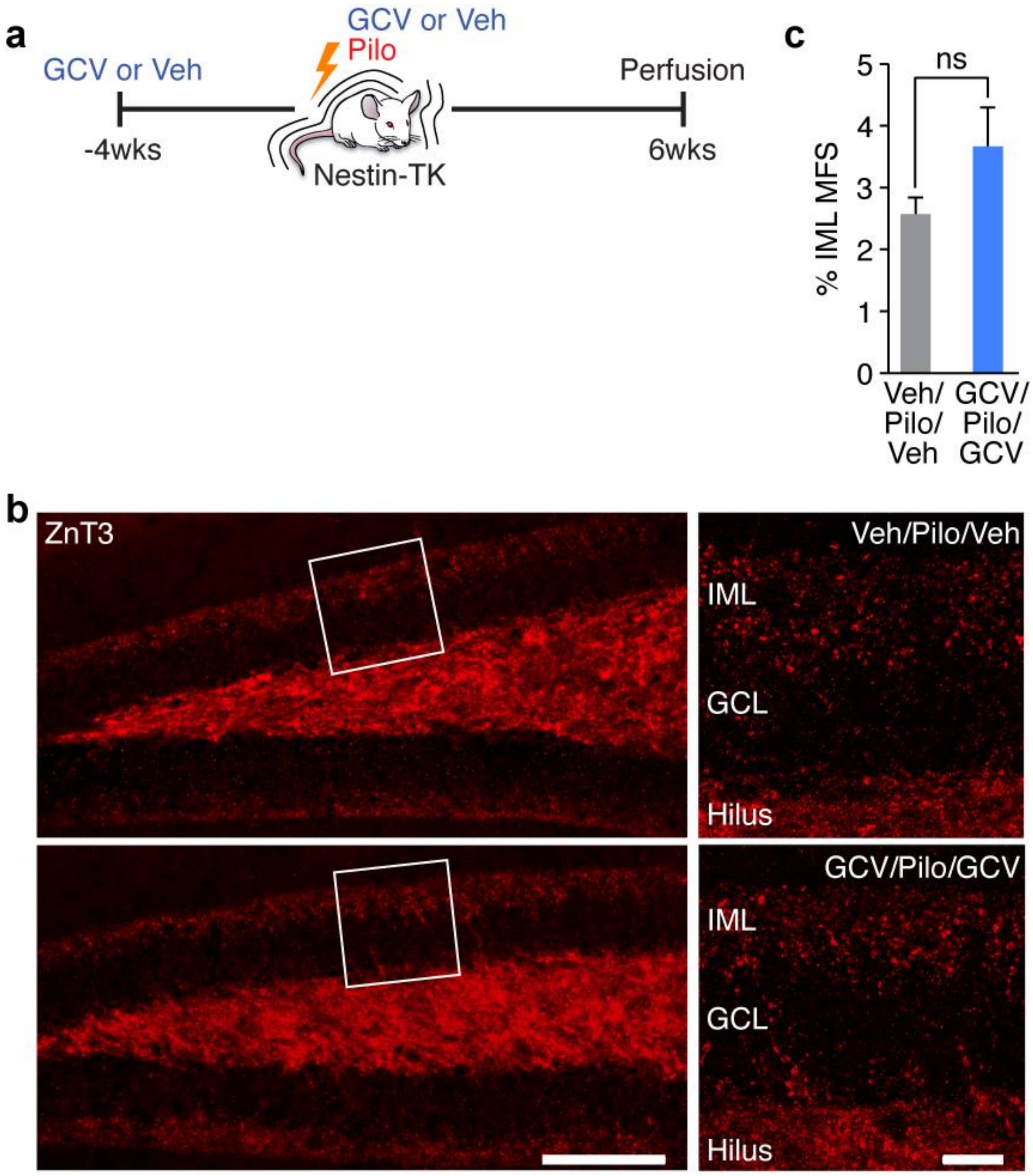

Supplementary Figure 4 | Near complete ablation of neurogenesis does not affect MFS (a) Time line to show experimental design. **(b)** Representative microscopic images from three independent experiments showing hippocampal zinc transporter-3 (ZnT3) immunoreactivity, a marker for the axons of granule cells, in epilepsy. Scale bar is 100 μm. Higher magnification images of ZnT3 immunoreactivity are shown in the right panel. Scale bar is 20 μm. **(c)** A graph measuring ZnT3-positive areas in IML of the dentate gyrus between Veh/Pilo/Veh (n =11) and GCV/Pilo/GCV (n = 13). Student's t-test, P = 0.148, t(22) = -1.501. Data present as mean ± s.e.m. ns: not significant. GCV, ganciclovir; Veh, vehicle; Nestin-TK, Nestin-δ-HSV-thymidine kinase-EGFP transgenic mice; Pilo, pilocarpine; IML, inner molecular layer; GCL, granule cell layer; MFS, mossy fiber sprouting.

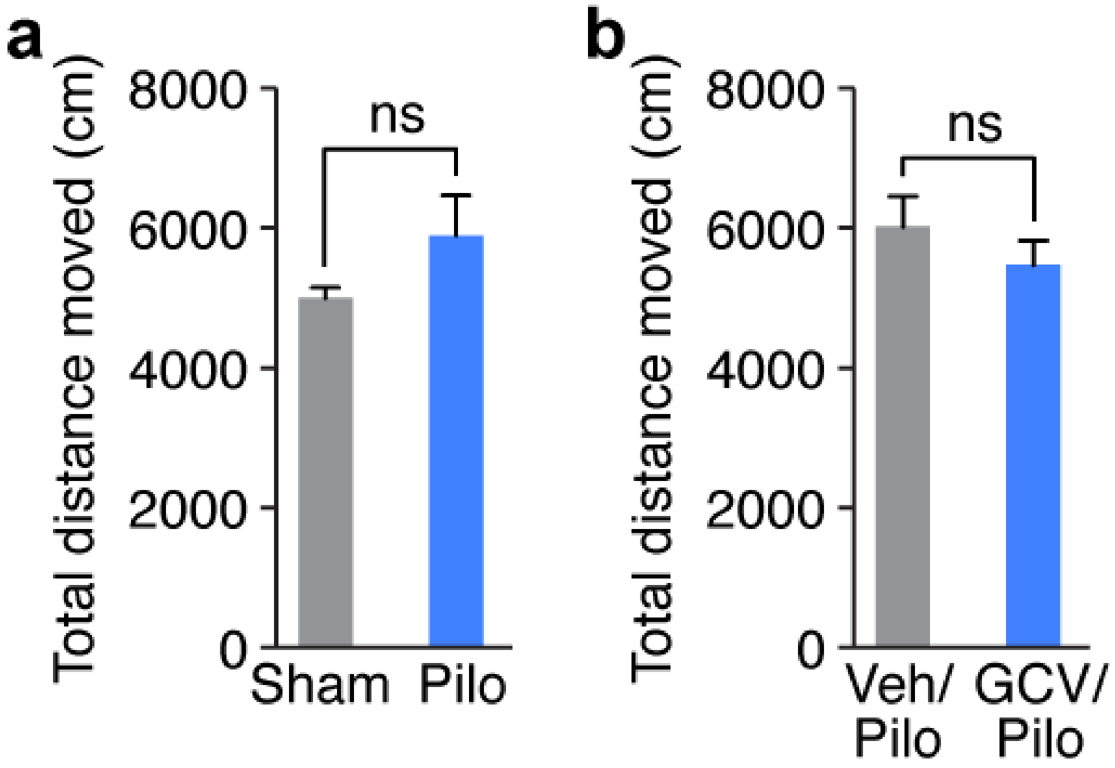

Supplementary Figure 5 | No change in locomotor activity (a) A graph showing total distance moved in open field test between sham (n = 11) and pilocarpine (Pilo)-injected groups (n = 12). Student's t-test, P = 0.156, t(12.518) = -1.508. **(b)** A graph showing total distance moved in open field test between Veh/Pilo (n = 19) and GCV/Pilo groups (n = 18). Mann-Whitney U test, P = 0.114, U = 119.000. Data present as mean ± s.e.m. ns, not significant. GCV, ganciclovir; Veh, vehicle.

Supplementary Methods

Mice *NeuroD*^{loxP}[1], and Nestin-CreER^{T2}; R26R-YFP mice[2] were genotyped by PCR using genomic DNA and primers for Cre (5'-GGT CGA TGC AAC GAG TGA TGA GG-3', 5'-GCT AAG TGC CTT CTC TAC ACC TGC G-3'), R26R-reporter (5'-AAA GTC GCT CTG AGT TGT TAT-3', 5'-GCG AAG AGT TTG TCC TCA ACC-3', 5'-GGA GCG GGA GAA ATG GAT ATG-3'), and *NeuroD* (5'-GTT TTT GTG AGT TGG GAG TG-3', 5'-TGA CAG AGC CCA GAT GTA-3'). *Nestin-CreER*^{T2}*; NeuroD*$^{loxP/+}$ mice were crossed with *NeuroD*$^{loxP/+}$*; R26R-YFP* mice to generate *Nestin-CreER*^{T2}*;NeuroD*$^{loxP/loxP}$*;R26R-YFP* (cKO) and *Nestin-CreER*^{T2}*;NeuroD*$^{+/+}$*;R26R-YFP* (wild-type; WT) mice. Mice at 5 weeks of age were administered tamoxifen (TAM; i.p.) at 150 mg kg^{-1} d^{-1} for 5 days, which was dissolved in 10% EtOH/90% sunflower seed oil.

Chemo-convulsant models of temporal lobe epilepsy For NeuroD WT or cKO mice, mice at 6 weeks of age were intraperitoneally injected with kainic acid (KA; Cayman Chemical 78050) dissolved in normal saline at 25 mg kg^{-1} to induce acute seizures. Seizure activity was behaviorally monitored according to the modified Racine's scale[3]. At 30 min after the initial KA injection, mice were injected with additional KA (A.G. Scientific K1013) at 5 mg kg^{-1} if their seizures were milder than stage 3. Mice exhibiting multiple stage 5 seizures were selected for further experiments. Moist chow, saline, and 5% dextrose solution were provided if necessary to facilitate recovery. Three weeks later, mice were perfused and processed for immunohistochemistry. For sham, all the procedures were the same except saline was injected instead of KA.

Immunohistochemistry Primary antibodies in this study were chosen based on the validation results by the manufacturer: goat anti-NeuroD (1:1,000, Santa Cruz Biotechnology sc-1084), chicken anti-GFP (used for GFP or YFP detection; 1:8,000, Aves Lab GFP-1020), guinea pig anti-DCX (1:2,000, Millipore AB2253), rabbit-anti-Prox1 (1:500, Millipore AB5475), rabbit-anti-ZnT3 (1:300, a gift from Dr. Palmiter), rabbit anti-Ki67 (1:500, Thermo Scientific RM-9106-S). For double or triple labeling, primary antibodies were simultaneously incubated (e.g., YFP/DCX/Ki67, YFP/Prox1) and further processed for each antibody. For NeuroD, DCX, Ki67, ZnT3, and Prox1, a fluorescent-tagged secondary antibody was used (1:300, Jackson ImmunoResearch). For YFP, primary antibody incubation was followed with an appropriate biotin-tagged secondary antibody (1:200, Jackson ImmunoResearch) for 1 h at room temperature followed by ABC (Vector Laboratories PK-6100) for 1 h and Tyramide-Plus signal amplification (1:50, PerkinElmer NEL701001KT) for 10 min. Sections were counterstained with DAPI (4,6-diamidino-2-phenylindole; 1:5,000, Roche 236276).

Microscopic analysis and quantification For MFS analysis, a minimum of 4 sections from each animal was measured. Pixel area of the dentate gyrus, including the granule cell layer and molecular layer but excluding the hilus was measured using NIH ImageJ software. The pixel intensity of the molecular layer for each section was measured as the background intensity. Using the threshold function, the area of pixels greater than the background intensity was measured as ZnT3 staining area. Data is reported as percentage of ZnT3 staining area to the area of the dentate gyrus.

Behavioral tests Behavioral tests were conducted from 5 to 7 weeks after pilocarpine or saline injection, starting with open field test followed by novel location (NL) and novel object (NO) recognition tasks. Locomotor activity in the open field box was assessed during the 15 min habituation phase without objects in the NL task (Day 1). Mice were positioned in the center of the box, and then individual total distance

moved during the first 10 min of habituation was automatically recorded using video tracking system (Noldus Information Technology).

Statistics All of the data are expressed as mean ± standard error of the mean (s.e.m.). Experimental groups were assigned by simple randomization and data was collected blind. SPSS (version 21.0, IBM SPSS Corp.) software was used for statistical comparison. Statistical differences were analyzed using two-tailed Student's *t*-test for the data with equal variances (Fig. S3f, S3h left, S4c) or Student's *t*-test with Satterthwaite's correction for the data with unequal variances (Fig. S5a). If normal distribution was not assumed, Mann-Whitney U test was performed (Fig. S1c, S2c, S3c left, S3c right, S3h right, S5b). Values of $P < 0.05$ were considered significant.

Supplementary References

1. Goebbels, S. *et al.* Cre/loxP-mediated inactivation of the bHLH transcription factor gene NeuroD/BETA2. *Genesis* **42**, 247-252 (2005).
2. Lagace, D.C. *et al.* Dynamic contribution of nestin-expressing stem cells to adult neurogenesis. *J. Neurosci.* **27**, 12623-12629 (2007).
3. Racine, R.J. Modification of seizure activity by electrical stimulation. II. Motor seizure. *Electroencephalogr. Clin. Neurophysiol.* **32**, 281-294 (1972).
